# Identification and Characterization of Piwi-Interacting RNAs for Early Testicular Development in Yak

**DOI:** 10.3390/ijms232012320

**Published:** 2022-10-14

**Authors:** Yongfu La, Xiaoming Ma, Pengjia Bao, Min Chu, Ping Yan, Xian Guo, Chunnian Liang

**Affiliations:** 1 Animal Science Department, Lanzhou Institute of Husbandry and Pharmaceutical Sciences, Chinese Academy of Agricultural Sciences, Lanzhou 730050, China; 2 Key Laboratory of Animal Genetics and Breeding on Tibetan Plateau, Ministry of Agriculture and Rural Affairs, Chinese Academy of Agricultural Sciences, Lanzhou 730050, China; 3 Key Laboratory for Yak Genetics, Breeding, and Reproduction Engineering of Gansu Province, Chinese Academy of Agricultural Sciences, Lanzhou 730050, China

**Keywords:** testis, piRNA, yak, small RNA sequencing, reproduction

## Abstract

Normal testicular development plays a crucial role in male reproduction and is the precondition for spermatogenesis. PIWI-interacting RNAs (piRNAs) are novel noncoding RNAs expressed in animal germ cells that form complexes with PIWI family proteins and are involved in germ cell development, differentiation, and spermatogenesis. However, changes in piRNA expression profiles during early testicular development in yak have not been investigated. In this study, we used small RNA sequencing to evaluate the differences and potential functions of piRNA expression profiles in 6-, 18-, and 30-month-old yak testis tissues. Differential expression analysis found 109, 293, and 336 differentially expressed piRNAs in M30 vs. M18, M18 vs. M6, and M30 vs. M6, respectively, and found 30 common differentially expressed piRNAs in the three groups of M6, M18, and M30. In addition, the functional enrichment analysis of differentially expressed piRNAs target genes indicated that they were related to testicular development and spermatogenesis. Finally, we detected the expression of the PIWI protein family in the yak testis at different developmental stages and found that *PIWIL1*, *PIWIL2*, *PIWIL3*, and *PIWIL4* were highly expressed in 18- and 30-month-old yak testis and almost not expressed in 6-month-old yak testis. In conclusion, this study summarizes the changes of piRNA expression patterns during the early development of yak testis and provides new clues for the regulatory role of piRNA in yak testis.

## 1. Introduction

The yak lives in the plateau area centered on the Qinghai–Tibet Plateau, at an altitude of 2000–5000 m. It can provide leather, transportation, milk, and meat for the area. It is the main dietary raw material and an important source of economic income for the local Tibetan people [1]. Compared with other livestock, the limitations of the plateau environments, economic underdevelopment, and the limitations of scientific approaches have limited the development of the yak industry. Reproduction is the key to improving yak production performance; in particular, the improvement of male yak breeds should not be ignored [2]. Early in testicular development, the somatic cells in bipotential gonads begin to differentiate into Sertoli cells and guide germ cells to the spermatogenic lineage. Normal differentiation and maintenance of Sertoli cells are critical to male reproductive health, and their numbers determine sperm production capacity. Androgens secreted by Leydig cells are important for male reproductive health and testicular development [3]. Therefore, it is of practical significance to understand and master the reproductive physiological characteristics of the early development of yak testis.

PIWI-interacting RNA (piRNA) is an under-studied small non-coding RNA with a length of about 21–32 nucleotides, which was first discovered in the asymmetry division of Drosophila germline stem cells and specifically binds to members of the PIWI protein family [4,5,6]. piRNAs exhibit a strong uracil bias at the 5′ end, arising from transposable element (TE) sequences or genomic clusters that are completely unrelated to TE, and their mechanism of action is by interacting with their target mRNAs, which are then captured by the the endonuclease activity of PIWI proteins [7,8,9]. In recent years, multiple studies have shown that piRNAs have functions in the regulation of gene expression, and several recent studies have revealed new functions for piRNAs in germ cell specification, the physiological regulation of spermatogenesis and germline development, embryonic patterning, the regulation of mRNAs and long noncoding RNAs, and genome protection from transposons [4,10,11]. In male mouse germ cells, the piRNA-guided post-transcriptional regulation of transposon mRNA is essential for chromatin and transcriptional landscape changes during meiosis [12,13]. In adolescent rats, testosterone may activate the piRNA and PIWI proteins, thereby affecting testicular function by mediating rat piRNA expression [14]. However, little is known about the expression patterns of smallRNAs in the yak testis and their regulatory roles during early testicular development.

Normal testicular development plays a crucial role in species breeding, and the study of early testicular development in yak is important to improve male yak semen quality and to promote the development of the yak industry. In addition, piRNAs play important roles in testicular development and spermatogenesis, but the specific functions and regulatory mechanisms of piRNAs remain unknown in yak testis. To investigate the relationship between piRNAs and yak testis development and spermatogenesis, we performed smallRNA sequencing to detect piRNAs expression profiles of yak testis at different developmental stages. Our study will provide a good model for studying the mechanisms that regulate testicular development and spermatogenesis and give us newer insights regarding the regulation of male yak reproduction.

## 2. Results

### 2.1. Overview of piRNA Expression Profiles in Yak Testis

**Figure 1:**
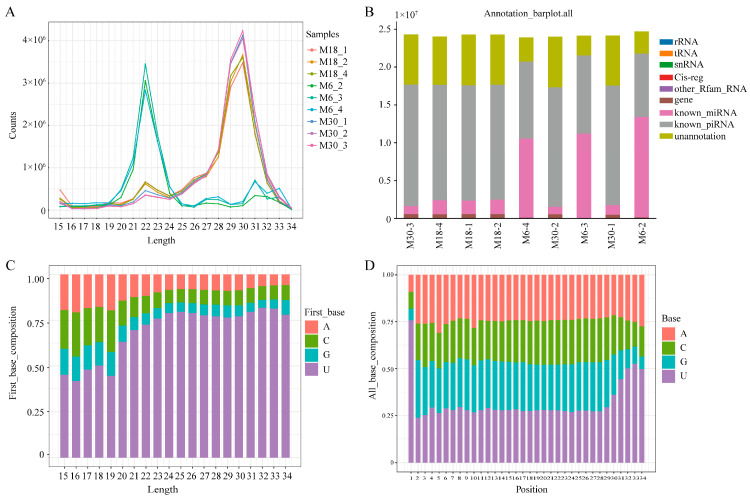
Characterization of piRNAs present in the yak testis. (**A**) Length distribution of the small RNAs identified with small RNA sequencing; (**B**) category of small RNA with uniq-clean reads from the small RNA sequencing; (**C**) visual diagram of the preference of the first base of piRNAs with different lengths; (**D**) visual diagram of base bias at each site of the piRNA sequence.

To identifiy yak piRNAs, smallRNA sequencing was performed on three groups of testis, from 6-, 18-, and 30-month-olds. To characterize the piRNAs, we first performed deep sequencing on the small RNAs ranging in length from 18 to 40 nt and identified 217.89 M unique reads from a total of 226.19 M reads. Small RNA analysis of mapped reads revealed two distinct peaks, one at 21–23 nt, corresponding to the size of the miRNA, and another at 29–31 nt, corresponding to the size of the piRNA (Figure 1A). For each sample, 240,104–1,858,245 reads were obtained, including miRNA, rRNA, tRNA, snRNA, Cis-reg, others, known_piRNA, and unannotated (Figure 1B). Sequence analysis of cloned small RNAs ranging from 18–40 nt in length showed a strong preference for adenine at position 10 and uridine at the 5′ end (Figure 1C,D). A total of 1,048,575 piRNAs were predicted and 1500 piRNAs were expressed in yak testis tissues (Table S1).

### 2.2. Differential Expression of piRNAs

**Figure 2:**
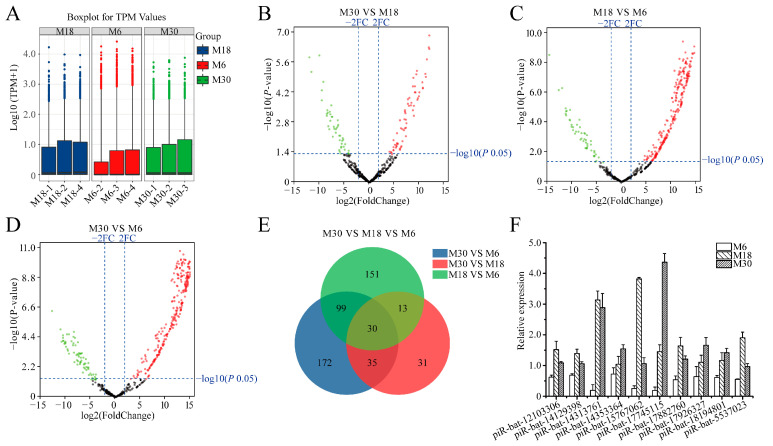
Comparative analysis of piRNAs in yak testis at different developmental stages. (**A**) The expression level of miRNAs in 6-, 18- and 30-month-old yak testes; (**B**) differences of piRNA expression between M30 and M18 libraries; (**C**) differences of piRNA expression between M18 and M6 libraries; (**D**) differences of piRNA expression between M30 and M6 libraries; (**E**) common differentially expressed piRNAs of M30, M18 and M6 libraries; (**F**) validation of piRNAs expression levels with real-time PCR; red, green, and grayness dots in the graph represent transcripts that were significantly upregulated, downregulated, and unchanged, respectively.

Whole testes were decapsulated and epididymides, fat, and fascia removed before total RNA extraction. All testicular samples were transported on dry ice to Shanghai OE Biotech Co., Ltd. (Beijing, China) for total RNA extraction. To investigate differentially expressed piRNAs during yak testicular development, differential expression analysis of the identified piRNAs was performed using the DESeq method. The expression of piRNAs in the M6, M18, and M30 groups was measured based on transcripts per million, which showed no abnormal expression in the nine samples (Figure 2A). A total of 109 DE piRNAs were identified from M30 and M18, including 63 upregulated and 46 downregulated piRNAs (Figure 2B and Table S2). A total of 293 DE piRNAs were identified from M18 and M6, including 243 upregulated and 50 downregulated piRNAs (Figure 2C and Table S3). A total of 336 DE piRNAs were identified from M30 and M6, including 265 upregulated and 71 downregulated piRNAs (Figure 2D and Table S4). There were 30 common differentially expressed piRNAs in the three developmental stages, M6, M18, and M30, in this study (Figure 2E and Table S5). To verify the authenticity of these differentially expressed piRNAs in the sequencing data, we selected ten common differentially expressed piRNAs (piR-bta-12103306, piR-bta-14129398, piR-bta-14313761, piR-bta-14353364, piR-bta-15767062, piR-bta-17745115, piR-bta-17882760, piR-bta-17926327, piR-bta-18194801, and piR-bta-5537023) and performed reverse transcription, followed by real-time PCR (Figure 2F). The real-time quantitative PCR results were consistent with the sequencing data, indicating that the sequencing data were accurate and reproducible.

### 2.3. Target Prediction of Differentially Expressed piRNAs

To understand the potential roles of differentially expressed piRNAs, the mRNAs targeted by these differentially expressed piRNAs were predicted using the Miranda algorithm [15]. In total, 71,851, 132,813, and 173,247 target sites in 24,152, 30,980, and 32,282 target genes were predicted for 109, 293, and 336 piRNAs obtained from M30 vs. M18 and M18 vs. M6 and M30 vs. M6, respectively. For most differentially expressed piRNAs, there were multiple distinct target genes; however, for some differentially expressed piRNAs, only one target gene was identified. In addition, some target genes are targeted by multiple differentially expressed piRNAs. For instance, *PIWIL2* is targeted by piR-bta-14313761, piR-bta-14986790, piR-bta-10940349, piR-bta-1910198, piR-bta-2029742, piR-bta-3158827, and piR-bta-640442; piR-bta-11096392 putatively targeted 342 mRNAs.

### 2.4. Functional Annotation of Differentially Expressed piRNAs

**Figure 3:**
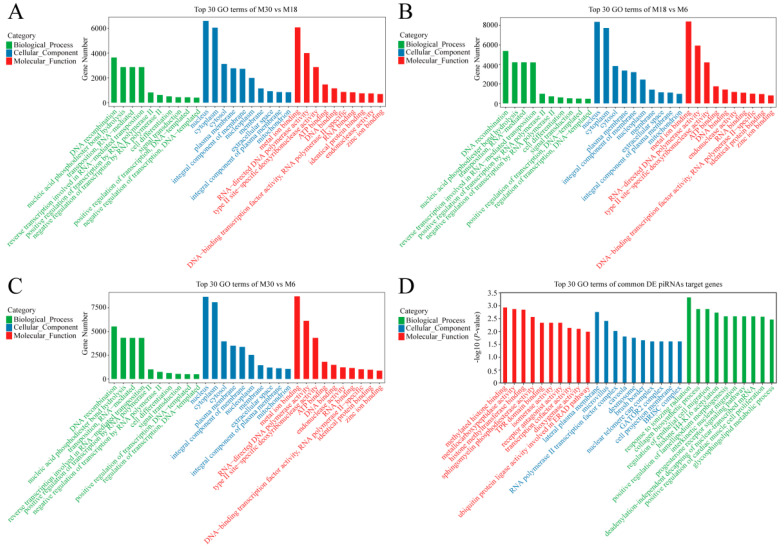
GO analysis of differentially expressed piRNA’s target genes. (**A**) GO enrichment analysis of differentially expressed piRNA’s target genes between M30 and M18; (**B**) GO enrichment analysis of differentially expressed piRNA’s target genes between M18 and M6; (**C**) GO enrichment analysis of differentially expressed piRNA’s target genes between M30 and M6; (**D**) GO enrichment analysis of common differentially expressed piRNA target genes.

In order to better understand the roles of differentially expressed piRNAs in the yak testicular development, GO and KEGG enrichment analyses were used. The GO analysis results are shown in Figure 3 and Tables S6–S9. During the testicular development, with similar top 10 biological processes, cellular components, and molecular functions, the top three GO processes of differentially expressed piRNAs included DNA recombination, nucleic acid phosphodiester bond hydrolysis, and transposition in the BP subgroup. Nucleus, cytoplasm, and cytosol were the top three processes in the CC subgroup. Metal ion binding, RNA directed DNA polymerase activity, and type II site-specific deoxyribonuclease activity were the top three processes in the MF subgroup (Figure 3A–C). The target genes of common differentially expressed piRNAs were involved in biological processes such as the deadenylation-independent decapping of nuclear-transcribed mRNA, glycosphingolipid metabolic process, cell fate commitment, and androgen receptor signaling pathway (Figure 3D).

**Figure 4:**
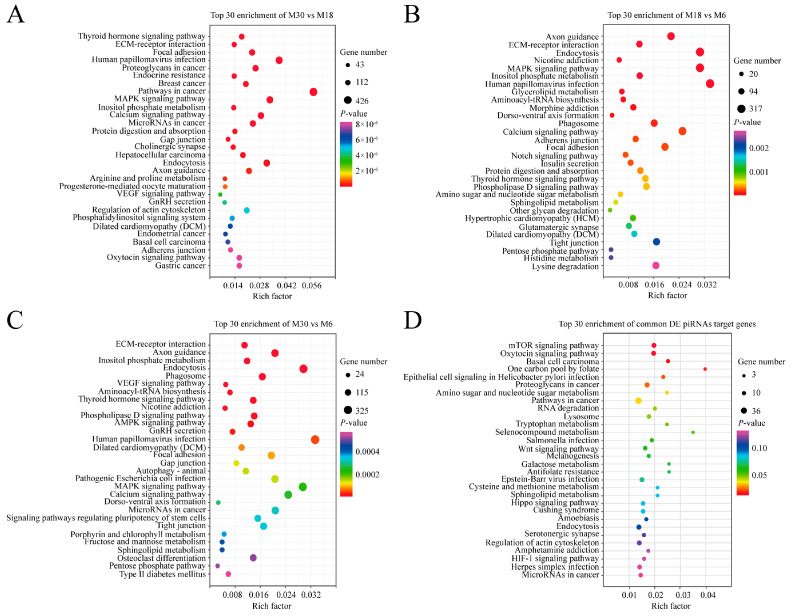
KEGG analysis of differentially expressed piRNA’s target genes. (**A**) KEGG analysis of differentially expressed piRNA’s target genes between M30 and M18; (**B**) KEGG analysis of differentially expressed piRNA’s target genes between M18 and M6; (**C**) KEGG analysis of differentially expressed piRNA’s target genes between M30 and M6; (**D**) KEGG analysis of common differentially expressed miRNA target genes; the vertical and horizontal axes represent the enrichment pathways and rich factors of these pathways, respectively. P-values of each pathway are color-coded.

KEGG pathway annotation showed that the target genes of differentially expressed piRNAs in the M30 and M18 comparison groups were significantly enriched in 131 pathways, such as the MAPK signaling pathway, GnRH secretion, PI3K-Akt signaling pathway, Aldosterone synthesis and secretion, GnRH signaling pathway, and Wnt signaling pathway (Figure 4A and Table S10). Between M18 and M6, the target genes were significantly enriched in 41 pathways, such as Axon guidance, MAPK signaling pathway, aldosterone synthesis and secretion, inositol phosphate metabolism, and insulin secretion (Figure 4B and Table S11). Between M30 and M6, the target genes were significantly enriched in 53 pathways, such as VEGF signaling pathway, AMPK signaling pathway, GnRH secretion, MAPK signaling pathway, and ABC transporters (Figure 4C and Table S12). The target genes of common differentially expressed piRNAs were enriched in the mTOR signaling pathway, RNA degradation, Wnt signaling pathway, and AMPK signaling pathway (Figure 4D and Table S13). The four enriched pathways related to testicular development that we focused on included AMPK, MAPK, PI3K-Akt, and the Wnt signaling pathway.

### 2.5. PIWIL Family Gene Expression in M6, M18, and M30 Yak Testis

**Figure 5:**
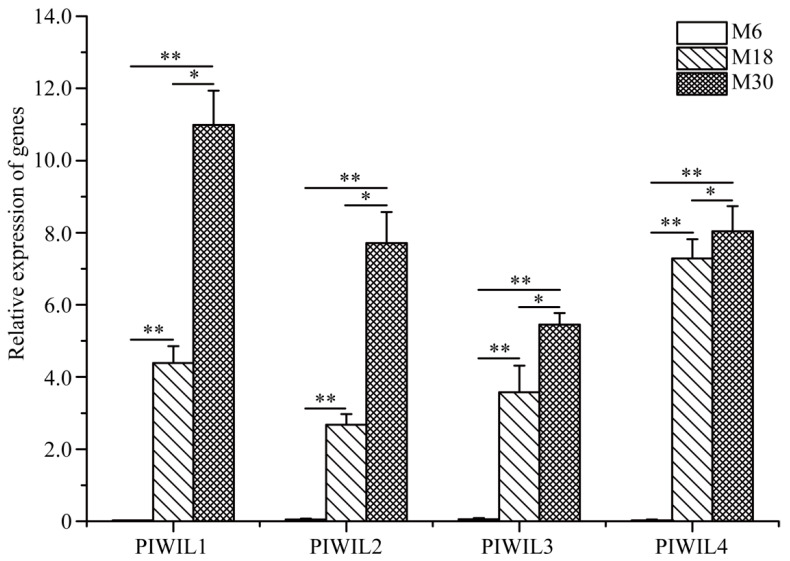
Relative mRNA expression of PIWIL family proteins was measured by qRT-PCR in 6-, 18-, and 30-month-old yak testes. * *p* < 0.05, ** *p* < 0.01.

PiRNAs play important regulatory roles in maintaining mRNA stability and genome structure, as well as in protein synthesis by binding to members of the PIWI family. Therefore, we examined the expression of yak PIWL family proteins in 6-, 18- and 30-month-old yak testis tissue (Figure 5). It was found that the expression of *PIWIL1*, *PIWIL2*, *PIWIL3*, and *PIWIL4* was higher in 18- and 30-month-old yak testis, and the expression of the *PIWI* family was nearly undetectable in 6-month-old yak testis. Moreover, the expression of *PIWIL1*, *PIWIL*2, *PIWIL3*, and *PIWIL4* was significantly higher in 30-month-old yak testis than in 18-month-old yak testis.

## 3. Discussion

The expression of proteins during the development of the testes is important to establishing fertility in the adult animal. Studies have shown that many genes are involved in the normal process of testicular development and spermatogenesis, but when these genes are mutated, they can cause spermatogenic disorder and infertility. PIWI-interacting RNAs are a novel class of noncoding RNAs that regulate germline development and gametogenesis by interacting with PIWI family proteins that are specifically expressed in animal germ cells [16,17,18]. Since the discovery of piRNAs and its function in regulating gene expression, many scholars have further studied piRNAs and found that piRNA is involved in multiple processes of testicular development and spermatogenesis [19,20,21,22]. However, few studies have been conducted to characterize piRNAs involved in the testicular development of early-puberty yaks. In this study, Illumina HiSeq X Ten technology was used to sequence small RNAs in the testicular tissues of 6-, 18- and 30-month-old male yak, analyze differentially expressed piRNAs, and perform GO enrichment and KEGG pathway analysis of the target genes in all libraries.

To investigate the gaps in the study of piRNA in the yak testis and the correlation between piRNA and testicular development, the yak testis tissue was sequenced, and the results were preliminarily analyzed. To our surprise, the number of differentially expressed piRNAs in all comparison groups was the most in M30 VS M6, followed by M18 VS M6, and the least in M30 VS M18, which is consistent with our predictions that yaks at 18 months of age tended to sexual maturity. There are a large number of differentially expressed piRNAs between the two comparison groups, but further validation and functional studies of these differentially expressed piRNAs are required. Preliminary analyses suggest that piRNAs in yak testis may regulate testicular development and are associated with the expression of testis-specific genes. Our results show that piRNAs may play important roles in yak testis and may be involved in regulating yak testicular development and spermatogenesis.

To understand the length and base preference characteristics of piRNAs in yak testis, we analyzed the length and base preference of the identified piRNAs and found that they were the same as those reported in other species. The first U and tenth A base reference reflects the endonuclease specificity and ping-pong pathway biogenesis, respectively [23]. In addition, most of the piRNAs were located in the repetitive sequence regions of the genome, indicating the function of yak piRNAs in post-transcriptional gene and transposon silencing, which is another character of piRNAs [24,25]. According to our analysis, the genomic localization and sequence characteristics of piRNAs in yak testis were very similar to those of other species, suggesting that piRNAs are evolutionarily conserved.

To gain a deeper understanding of the functions of piRNAs in the yak testis, we performed GO and KEGG enrichment analysis on the target genes of these differentially expressed piRNAs. GO enrichment analysis found that the target genes of these differentially expressed piRNAs were enriched in biological processes, such as DNA recombination, nucleic acid phosphodiester bond hydrolysis, and transposition. KEGG enrichment analysis found that the target genes of these differentially expressed piRNAs were significantly enriched in the AMPK, MAPK, PI3K-Akt, and Wnt signaling pathways related to testicular development and spermatogenesis. The AMPK signaling pathway regulates energy metabolism, cell proliferation, and junctional complex stability in Sertoli cells, and the disruption of this pathway affects sperm quality and testicular microenvironment [26,27]. The common differentially expressed piRNAs piR-bta-14313761, piR-bta-14353364, piR-bta-15348752, piR-bta-17745115, piR-bta-15567574, and piR-bta-17882760 target multiple members enriched in the AMPK signaling pathway, such as *PPP2R5A*, *IRS1*, *CCNA2*, *STRADB*, and *PRKAA2*, especially piR-bta-14313761, and target multiple genes in the AMPK signaling pathway, indicating that these piRNAs may play important roles in Sertoli cell proliferation and the maintenance of sperm quality. Evidence accumulated over the past decade has demonstrated the major functions of the MAPK, AMPK and PI3K-Akt signaling pathways during testicular development, with the MAPK and AMPK signaling pathways regulating germ cell and Sertoli cell proliferation and germ cell meiosis and the PI3K-Akt signaling pathway affecting the proliferation, survival and apoptosis of Leydig cells [28,29,30,31,32]. The Wnt signaling pathway components *WNTs*, *NKD1*, *FZD9*, and *CTNNB1* have been reported to be expressed in adult or developing testis, suggesting that WNT signaling could play roles in several testicular processes [33,34,35]. For the regulation of target genes in pathways related to testicular development and spermatogenesis, the most functional piRNAs were piR-bta-14313761, piR-bta-15125870, and piR-bta-15767062. Each of them regulated seven to twelve genes in the AMPK, MAPK, PI3K-Akt, and Wnt signaling pathways. It suggests that these piRNAs may be effective in testicular development and spermatogenesis.

PIWI family proteins form the PIWI-piRNA complex with piRNAs, which play important roles in regulating the expression of other genes [23,36,37]. In mammals, the PIWI family members, including *PIWIL1*, *PIWIL2*, *PIWIL3* and *PIWIL4*, are mainly expressed in germ cells and are essential for male fertility and spermatogenesis, and it is required for the expression of target mRNAs involved in spermatogenesis [38,39,40]. Therefore, we also wonder whether there were differences in the expression of PIWI family proteins during the early development of yak testis. Our results showed that the expression of *PIWIL1*, *PIWIL2*, *PIWIL3*, and *PIWIL4* was significantly different in M6, M18, and M30. Furthermore, *PIWIL1*, *PIWIL2*, *PIWIL3*, and *PIWIL4* seemed not to be expressed in M6. This may suggest that piRNAs in yak testis mainly performs their function during spermatogenesis by interacting with *PIWIL1*, *PIWIL2*, *PIWIL3*, and *PIWIL4*.

## 4. Materials and Methods

### 4.1. Ethics Statement

The study was approved by the Animal Administration and Ethics Committee of the Lanzhou Institute of Husbandry and Pharmaceutical Sciences of the Chinese Academy of Agricultural Sciences and Pharmaceutical Sciences and met the requirement of the institutional animal care and use committee (Permit No. 2019-002).

### 4.2. Animals and Sample Preparation

Three 6- (M6), 18- (M18), and 30-month-old (M30) male yaks were selected from the Ashidan yak nucleus herds at the Datong Breeding Farm of Qinghai Province. Testes were obtained by veterinary surgery, and experimental samples were obtained after the excision of the caudal epididymis, fat, and fascia around the testis. All samples were snap-frozen in liquid nitrogen (−196 °C), shipped to the laboratory, and stored at −80 °C for total RNA extraction.

### 4.3. Small RNA Sequencing

All testis samples were transported on dry ice to Shanghai Oebiotech (China), and the total RNA was extracted from three individuals in the M6, M18, and M30 groups using Trizol reagent (Invitrogen, Waltham, MA, USA), and small RNA sequencing was performed on an Illumina HiSeq X Ten. Sequencing libraries were then sequenced on an Illumina HiseqTM 2500 (Illumina Corp., San Diego, CA, USA) instrument to generate 150 nt single-end reads. Then, the raw data was checked using FASTQC tools. Using the Cutadapt (version 1.14., Dortmund, Germany) software to remove adapter sequences, filter low-quality reads, reads with 5′ primer contaminants and poly (A), the reads without 3′ adapter and inserted tags, and reads shorter than 15 nt or longer than 41 nt were filtered to obtain clean reads [41]. NGSQCToolkit (version 2.3.3., New Delhi, India) software was first used for adapter removal, and then low-quality bases and N-bases or low-quality reads were filtered out [42]. Finally, we obtained high-quality clean reads. Raw data for small RNA-seq have been documented in the SRA public database (Accession number: SRP390350).

### 4.4. Bioinformatics Analysis

To classify and annotate the small RNAs in the sequencing results, the clean reads were aligned and subjected to blast search against the Rfam (version 10.0., St Louis, USA), with E ≤ 0.01, miRBase, and the piRBase database [43,44,45]. The reads filtered by the above several methods to retain the 18–34 nt sequence; the obtained clean reads were aligned with the piRNA from the piRBase with Bowtie software without mismatches, and the sequence on the alignment are considered to be known piRNA [46]. Expression levels were calculated for known piRNAs, and transcripts per million (TPM) were used to calculate the expression levels of these known piRNAs [47]. Differentially expressed piRNAs were identified with the thresholds of *p*-value < 0.05 and fold change ≥ 2, using the DEG algorithm in the R package to calculate the *p*-values [48]. The targets of differentially expressed piRNAs were predicted by using the Miranda software in yak, with the parameters as follows: single-residue-pair match scores ≥ 150, ΔG ≤ −30 kcal/mol, and demand strict 5′ seed pairing [15]. Gene ontology (GO) enrichment and Kyoto Encyclopedia of Genes and Genomes (KEGG) pathway enrichment analysis of different expressed piRNA-target-genes were, respectively, performed using R based on the hypergeometric distribution (*p* ≤ 0.05).

### 4.5. Real-Time Quantitative PCR

Quantification was performed with a two-step reaction process: reverse transcription (RT) and PCR. Each RT reaction consisted of 0.5 μg RNA, 5 μL of 2 × TS miRNA reaction mix, and 0.5 μL of TransScrip miRNA RT Enzyme Mix, in a total volume of 10 μL. Reactions were performed in a GeneAmp^®^ PCR System 9700 (Applied Biosystems, Waltham, MA, USA) for 60 min at 37 °C, followed by the heat inactivation of RT for 5 s at 85 °C. The 10 μL RT reaction mix was then diluted ×10 in nuclease-free water and held at −20 °C. Real-time PCR was performed using a LightCycler^®^ 480 II Real-time PCR Instrument (Roche, Basel, Switzerland) with a 10 μL PCR reaction mixture that included 1 μL of cDNA, 5 μL of 2 × PerfectStartTM Green qPCR SuperMix, 0.2 μL of universal primer, 0.2 μL of microRNA-specific primer, and 3.6 μL of nuclease-free water. Reactions were incubated in a 384-well optical plate (Roche, Basel, Switzerland) at 94 °C for 30 s, followed by 45 cycles of 94 °C for 5 s and 60 °C for 30 s. Each sample was run in triplicate for analysis. At the end of the PCR cycles, melting curve analysis was performed to validate the specific generation of the expected PCR product. Actin and U6 were selected as reference genes for the expression analysis of PIWIL family genes and piRNAs, respectively. The 2^−ΔΔCt^ method was used to analyze Ct values [49]. Primer sequences are shown in Table S14.

## 5. Conclusions

To sum up, in light of our understanding, we are the first to search for piRNAs in 6-, 18-, and 30-month-old yak testis tissue by a high-throughput method. We detected the expression of piRNAs in the early yak testis and discovered the differentially expressed piRNAs in 6-, 18-, and 30-month-old yak testis by a high-throughput method. Then, we predicted the functions of these differentially expressed piRNAs target genes and found that piRNAs in yak testis may regulate testicular development and spermatogenesis through a miRNA-like post-transcriptional gene silencing way. Finally, we detected the expression of the PIWI protein family in the yak testis at different developmental stages, laying a foundation for the further study of piRNA expression in yak testis. Our results may help to further understand the role of piRNAs in yak testicular development, increased our further understanding of the mechanism of testicular development and spermatogenesis, and also laid some foundations for further research on the mechanism of piRNAs in testis development and spermatogenesis.

## Data Availability

The data presented in this study are openly available in the SRA public database (Accession number: SRP390350).

## Supplementary Materials

The following supporting information can be downloaded at: https://www.mdpi.com/article/10.3390/ijms232012320/s1.

## Abbreviations

piRNA: PIWI-interacting RNA; DE: differentially expressed; GO: gene ontology; KEEG: Kyoto Encyclopedia of Genes and Genomes; qRT-PCR: quantitative real-time PCR; M6: 6-month-old; M18: 18-month-old; M30: 30-month-old; TPM: transcripts per million.
